# Design and Calibration of a Novel Bio-Inspired Pixelated Polarized Light Compass

**DOI:** 10.3390/s17112623

**Published:** 2017-11-14

**Authors:** Guoliang Han, Xiaoping Hu, Junxiang Lian, Xiaofeng He, Lilian Zhang, Yujie Wang, Fengliang Dong

**Affiliations:** 1 College of Mechatronics and Automation, National University of Defense Technology, Changsha 410073, China; hanguoliang09@nudt.edu.cn (G.H.); jx_lian@hotmail.com (J.L.); hexiaofeng@nudt.edu.cn (X.H.); lilianzhang@nudt.edu.cn (L.Z.); yjwang@nudt.edu.cn (Y.W.); 2 Nanofabrication Laboratory, Key Laboratory of Nanosystem and Hierarchical Fabrication, CAS Center for Excellence in Nanoscience, National Center for Nanoscience and Technology, Beijing 100190, China; dongfl@nanoctr.cn

**Keywords:** polarization navigation, skylight polarization pattern, pixelated polarizer array, sensor calibration, Rayleigh scattering

## Abstract

Animals, such as Savannah sparrows and North American monarch butterflies, are able to obtain compass information from skylight polarization patterns to help them navigate effectively and robustly. Inspired by excellent navigation ability of animals, this paper proposes a novel image-based polarized light compass, which has the advantages of having a small size and being light weight. Firstly, the polarized light compass, which is composed of a Charge Coupled Device (CCD) camera, a pixelated polarizer array and a wide-angle lens, is introduced. Secondly, the measurement method of a skylight polarization pattern and the orientation method based on a single scattering Rayleigh model are presented. Thirdly, the error model of the sensor, mainly including the response error of CCD pixels and the installation error of the pixelated polarizer, is established. A calibration method based on iterative least squares estimation is proposed. In the outdoor environment, the skylight polarization pattern can be measured in real time by our sensor. The orientation accuracy of the sensor increases with the decrease of the solar elevation angle, and the standard deviation of orientation error is $0.15^{\circ }$ at sunset. Results of outdoor experiments show that the proposed polarization navigation sensor can be used for outdoor autonomous navigation.

## 1. Introduction

Navigational ability is essential for a variety of human activities, and it is also an important guarantee for unmanned platforms to autonomously accomplish their tasks. Current navigation approaches are mostly dependent on global navigation satellite systems (GNSS) and inertial navigation systems (INS). However, the signals of GNSS are easily disturbed, and probably unavailable in many terrestrial environments such as near tall buildings and in forests. The error of INS will increase rapidly with time, so the accuracy of inertial navigation system cannot meet the long-term navigation needs (several hours or even several days) in terms of current technical levels.

The remarkable navigation ability that many animals possess provides a good reference for us. Small insects have excellent navigation capabilities, and, even in complex environments, their compound eyes are able to help them navigate effectively and robustly [1]. Many animals can derive compass information from the skylight polarization pattern that appears due to the scattering of sunlight in the sky. Homberg et al. [2] analyzed the polarization-vision pathways in locusts and crickets and found that the detection of sky polarized light depends on the special photoreceptor cells in the small dorsal rim area of the compound eye. Stevent et al. [3] explored sunlight-dependent parameters for navigation in North American monarch butterflies, and demonstrated that the polarization angle of sky polarized light is an important parameter for orientation. Muheim et al. [4] found that migrating Savannah sparrows derive an absolute direction from skylight polarization patterns at sunrise and sunset, and use it as the primary calibration reference to calibrate the magnetic compass. Like sunlight, moonlight scatters when it hits tiny particles and creates celestial polarization patterns. Dacke et al. [5] found that an African dung beetle can determine their own direction according to the skylight polarization pattern in the moonlight. The key to polarization navigation, whether in sunlight or in moonlight, is to extract the heading information from the skylight polarization pattern.

Many research groups have done plenty of work on measurements of skylight polarization patterns and methods of extracting compass information [6,7,8]. At present, the measurements of polarized light can be divided into two categories: single-point based and image based measurement modes. Single-point based navigation sensors use multiple photodiodes to extract polarization information of incident light from a certain direction. Lambrinos et al. [9] constructed a polarization compass using photosensitive diodes and successfully employed it for robot navigation. Chu and co-workers [10,11] analyzed the mechanism by which ants perceive the polarized light with compound eyes and proposed an estimation algorithm for angle of polarization. However, the point-source based sensors are extremely susceptible to environmental interference. In order to minimize the effect of noise and improve the robustness, image-based polarization measurements are exploited by researchers. Polarization imaging [12,13] is the process of recording the full or partial Stokes vectors of light across an image plane. Andreou et al. [14] first introduce the basic knowledge of polarization sensing and discuss a number of integrated sensory devices designed for polarization imaging. Pust and Shaw built an imaging Stokes-vector polarimeter to study the skylight polarization pattern [15]. Wang et al. [16] proposed a bionic polarization navigation sensor based on three cameras, which has two measurement modes mentioned above. Ren et al. [17] introduced a cluster analysis method to locate the sun and solar meridian from the skylight polarization pattern. Line detection methods have been widely used in vision based navigation [18,19]. Lu et al. [20] used Hough transform to extract solar meridian from points having a polarization angle of nearly $90^{\circ }$ in polarization imaging. Tang et al. [21] proposed a method based on Pulse Coupled Neural Network (PCNN) algorithm to calculate compass information from the polarized skylight imaging, and obtained good accuracy under harsh conditions. Based on a single scattering Rayleigh model, Wang et al. [22] designed a solar meridian detection algorithm taking into account the polarization information of all the pixels. However, the above image-based polarized light sensors are too complicated and bulky, so it is not easy for these sensors to be installed on small robot platforms. The rapid development of nanofabrication techniques promotes the research on division of focal plane (DoFP) imaging sensor, which integrates pixelated polarization filters and imaging elements to realize a real-time and high-resolution measurement of the incident polarization information [23,24,25,26,27]. The design and fabrication of polarizers [28,29,30,31] directly affect the performance of the sensor. York et al. [32] analyzed the factors that influence the estimation accuracy of angle and degree of polarization for a DoFP sensor, and then present a measurement system to quantify these effects. In order to mitigate the loss of spatial resolution due to subdivision of the array, Gao et al. [33] proposed a gradient based interpolation algorithm.

In this paper, a novel polarization sensor, which mainly consists of a Charge Coupled Device (CCD) camera, a wide-angle lens and a pixelated polarizer array, is proposed. We establish the error model, including the response error of CCD pixels and installation error of pixelated polarizer array, and then put forward the calibration method. Indoor and outdoor experiments show that our sensor can achieve very excellent orientation results, which prove the accuracy of the error model and the effectiveness of the calibration method. The advantages of the proposed sensor are as follows: firstly, compared with multi-camera based polarized light compass, our sensor is more integrated, with the advantages of having a small size and being light weight. Secondly, the sampling rate of the sensor can reach 80 frames per second because one image is enough to calculate the skylight polarization pattern.

The rest of this paper is organized as follows: Section 2 introduces the structure of the proposed sensor, the measurement method of the skylight polarization pattern and the orientation method. Section 3 establishes the error model and then proposes the corresponding calibration method. Section 4 presents results of indoor calibration and the outdoor experiment. Finally, conclusions are drawn in Section 5.

## 2. Materials and Methods

### 2.1. Design of Pixelated Polarized Light Compass

**Figure 1:**
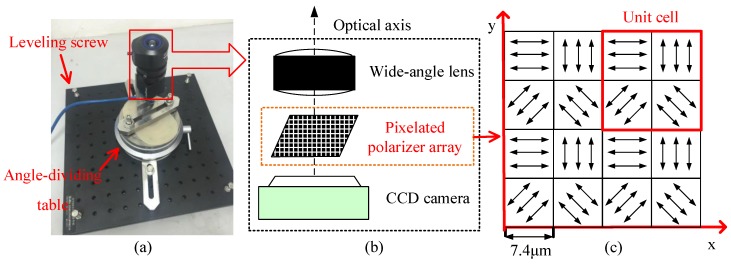
(**a**) the pixelated polarized light compass; (**b**) the installing structure of the sensor; (**c**) layout design of the pixelated polarizers.

The system composition of the designed pixelated polarized light compass (PPL-compass) is shown in Figure 1b. The PPL-compass is mainly composed of a CCD camera, a wide-angle lens that has small distortion and a pixelated polarizer array. The CCD camera (PointGrey, BFLY-U3-03S2M) has a resolution of 640 × 480. The focal length of the wide-angle lens is 3mm, and the field of view of which is about $77^{\circ }\times 61^{\circ }$. As shown in Figure 1c, the pixelated polarizer array based on aluminum nano-grating is composed of a 160 × 120 array of unit cells, each of which consists of a 2 × 2 array of polarizers. The adjacent four polarizers in one unit cell pass linearly polarized light along different directions. The top left-hand and the bottom left-hand polarizers pass linearly polarized light oriented along the *x*-axis and at 45${}^{\circ }$ counterclockwise to the *x*-axis respectively, while the polarization direction of the right two polarizers are perpendicular to that of the left two polarizers. The aperture of each polarizer is 7.4 μm × 7.4 μm, the size of which is exactly the same as the size of CCD camera pixels. The grating of each polarizer has a period of 140 nm and a duty cycle of 0.5, and the depth of the grating is 200 nm.

### 2.2. Measurement of Skylight Polarization Patterns

**Figure 2:**
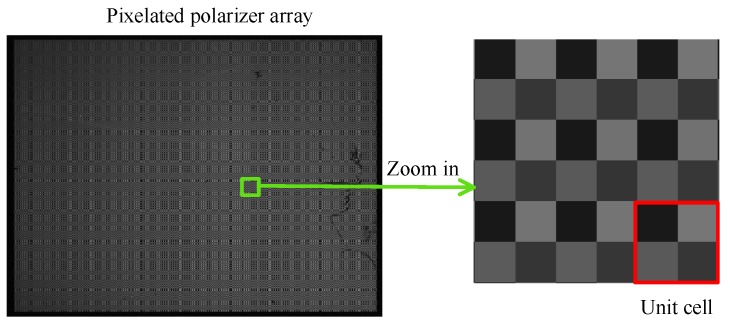
Intensity response of the Charge Coupled Device (CCD) pixels under linearly polarized light.

Figure 2 shows the intensity response of the CCD pixels under linearly polarized light, and the enlarged image contains multiple polarization measurement units. Since the incident light is linearly polarized, the responses of the four pixels in one measurement unit are not the same.

The four polarizers in one unit cell are very close to each other, so it can be assumed that the four polarizers receive the same incident light. The intensity of the light passing through the four polarizers can be described as:

(1) $$f_{j}=\frac{1}{2}I\left[1+d\cos\left(2\phi -2\alpha_{j}\right)\right],$$

where j=1,2,3,4 denotes the number of four polarizers in a unit cell, *I* is the intensity of incident light, *d* is the degree of polarization, and ϕ is the angle between the polarization direction of the incident light and the reference direction. $\alpha_{j}$ is the angle between the polarity direction of polarizers and the reference direction.

Equation (1) can be rewritten as:

(2) $$f_{j}=Id\cos2\phi \cos2\alpha_{j}+Id\sin2\phi \sin2\alpha_{j}+I.$$

Define

$$A=\left[\begin{array}{ccc} \cos2\alpha_{1} & \sin2\alpha_{1} & 1\\ \cos2\alpha_{2} & \sin2\alpha_{2} & 1\\ \cos2\alpha_{3} & \sin2\alpha_{3} & 1\\ \cos2\alpha_{4} & \sin2\alpha_{4} & 1 \end{array}\right],Q=\left[\begin{array}{c} Id\cos2\phi \\ Id\sin2\phi \\ I \end{array}\right],F=\left[\begin{array}{c} f_{1}\\ f_{2}\\ f_{3}\\ f_{4} \end{array}\right].$$

Then, Equation (2) can be described in matrix form:

(3) AQ=F.

Equation (3) can be solved by least square estimation [34]:

(4) $$Q={\left(A^{T}A\right)}^{-1}A^{T}F.$$

The angle of polarization and degree of polarization for incident light is given by:

(5) $$\phi =\frac{1}{2}\arctan\left(\frac{q_{2}}{q_{1}}\right),\;d=\frac{\sqrt{q_{1}^{2}+q_{2}^{2}}}{q_{3}},$$

where $q_{1}$, $q_{2}$ and $q_{3}$ are elements of *Q*.

Actually, $q_{1}$, $q_{2}$ and $q_{3}$ are corresponding to the elements of the Stokes vector $S_{1}$, $S_{2}$ and $S_{0}$, respectively [32]. In the polarized sky light, the circular polarizations can be neglected, and the fourth element of the Stokes vector $S_{3}$ is left out in this paper. The degree of polarization in this paper is referred to as the degree of linear polarization (DOLP) [32]. Theoretically, at least three polarizers with different polarization directions are capable of measuring the first three Stokes parameters, and we choose a four-orientation layout based on the design in the literature [24,28,30].

### 2.3. Orientation Method

A skylight polarization pattern is a common natural polarized phenomenon, and polarized light is due to the scattering of the natural light with particles and dust in the atmosphere. In a clear sky, scattering particles are mainly composed of atmospheric molecules, the size of which is much smaller than the wavelength of the incident light.

**Figure 3:**
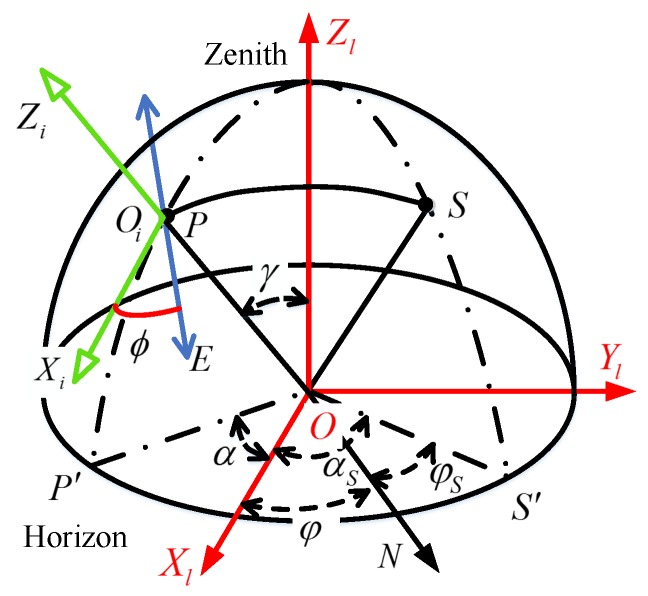
Description of the single scattering Rayleigh model.

The right-hand Cartesian coordinate frames shown in Figure 3 are defined as below:

$OX_{l}Y_{l}Z_{l}$: Camera coordinate frame. When the sensor is leveled, the axis $Z_{l}$ points to the zenith direction;

$O_{i}X_{i}Y_{i}Z_{i}$: Incident rays coordinate frame. The axis $Z_{i}$ points to the observation direction, the $X_{i}$ axis is in the plane $OPP^{\prime }$ and perpendicular to the observation direction (the axis $Y_{i}$ is not shown in Figure 3 for simplicity).

Figure 3 is the schematic diagram for the single scattering Rayleigh model. *O* represents the positon of observer, *S* is the position of the sun on the celestial sphere. The *N* axis is the geographic north, zenith angle and azimuth angle of the sun in the navigation coordinate frame is $\gamma_{S}$ and $\varphi_{S}$, respectively. *P* is the observation point on the celestial sphere. The zenith angle and azimuth angle of the observation point *P* in the camera coordinate frame are γ and α, respectively. The solar azimuth angle in camera coordinate frame is $\alpha_{S}$, and the polarization angle of scattered light is ϕ.

The expression of PE in frame *i* is:

(6) $$\vec{PE^{i}}={\left[\begin{array}{ccc} \cos\phi & \sin\phi & 0 \end{array}\right]}^{T}.$$

The transformation matrix $C_{i}^{l}$ from frame *i* to frame *l* is expressed as:

(7) $$C_{i}^{l}=\left[\begin{array}{ccc} \cos\alpha & -\sin\alpha & 0\\ \sin\alpha & \cos\alpha & 0\\ 0 & 0 & 1 \end{array}\right]\left[\begin{array}{ccc} \cos\gamma & 0 & \sin\gamma \\ 0 & 1 & 0\\ -\sin\gamma & 0 & \cos\gamma \end{array}\right].$$

The direction of E-vector in frame *l* can be calculated as follows:

(8) $$e=\vec{PE^{l}}=C_{i}^{l}\vec{PE^{i}}.$$

The zenith angle γ and azimuth angle α of the observation point *P* in frame *l* can be calculated by the following formula:

(9) $$\begin{array}{c} \gamma =\arctan(\frac{\sqrt{{\left(x_{p}-x_{c}\right)}^{2}+{\left(y_{p}-y_{c}\right)}^{2}}}{f_{c}}),\\ \alpha =\arctan(\frac{y_{p}-y_{c}}{x_{p}-x_{c}}), \end{array}$$

where $f_{c}$ denotes the focal length of the camera, $\left(x_{c},\;y_{c}\right)$ is the image principal point, and each pixel $\left(x_{p},\;y_{p}\right)$ in the image is corresponding to an observation point *P* in the sky.

According to the single scattering Rayleigh model, the E-vector of the scattered light is perpendicular to the scattering plane, that is, the E-vector *e* are perpendicular to the vector of sun direction *s*:

(10) $$e^{T}s=0.$$

Thus, the sun direction vector can be estimated by at least two uncorrelated E-vectors. Define $E={\left[\begin{array}{ccc} e_{1} & \cdots & e_{N} \end{array}\right]}_{3\times N}$, where *N* is the number of pixels. Then, we have:

(11) $$E^{T}s=0.$$

In actual measurements, the optimal estimation of the sun vector *s* can be expressed as the following optimization problem:

(12) $$\min_{s}\left(s^{T}EE^{T}s\right),s.t.s^{T}s=1.$$

The eigenvector of $EE^{T}$ corresponding to its smallest eigenvalue is the optimal estimation of the sun vector. Theoretically, the sun vector can be obtained by at least two uncorrelated E-vectors. The estimation accuracy of the polarization pattern will directly influence the estimation accuracy of the sun vector. When the sky is partly obscured by leaves or tall buildings, the estimation of angle and degree of polarization in non-sky area can be greatly disturbed. In order to reduce the estimation error of the sun vector, a possible solution is to detect the sky region first and then estimate the sun vector [35]. The number of polarization measurement units for each frame is about 19,000. Then, the time for solving the optimization equation is about 1 ms in Matlab (R2015b).

Assuming $s^{*}$ is the eigenvector corresponding to the minimum eigenvalue of $EE^{T}$. Then, the estimated solar azimuth angle is:

(13) $$\alpha_{S}^{*}=arc\tan\left(\frac{s_{2}^{*}}{s_{1}^{*}}\right),$$

where are $s_{1}^{*}$ and $s_{2}^{*}$ are the first and second elements of $s^{*}$, respectively.

According to the astronomical ephemeris, after inputting the time and location, solar zenith angle $\gamma_{S}$ and azimuth angle $\varphi_{S}$ can be obtained. Then, the heading angle in geographic coordinate frame is given by:

(14) $$\varphi =\varphi_{S}-\alpha_{S}^{*}.$$

## 3. Error Modeling and Calibration Method of the Sensor

### 3.1. Response Error of CCD Pixels

**Figure 4:**
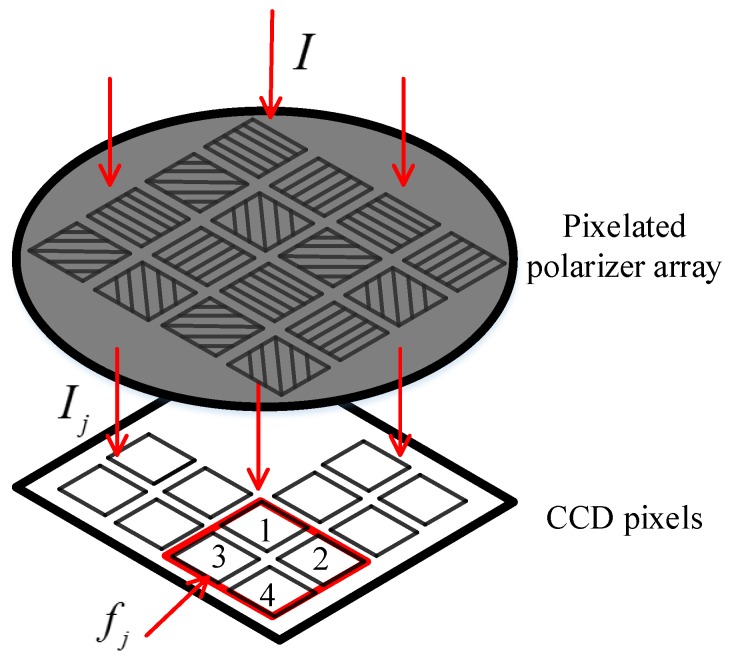
Schematic representation of the response of CCD pixels.

As shown in Figure 4, the incident light passes through the pixelated polarizer array and then irradiates on the CCD camera. Assuming that the incident light is natural light without polarization, the light intensity of the emitted light passing through the pixelated polarizer will be halved. Even if the intensity of the incident light is the same, the pixel responses of CCD camera are also inconsistent. This inconsistency depends mainly on the camera’s internal characteristics, the inconsistency of pixelated polarizers and noise. We can model the response of each pixel to the incident light intensity as a linear relationship. The linear response model contains the scale factor, bias and noise, where the measurement noise is white noise.

For a unit cell, the linear response model can be described by the following equation:

(15) $$f_{j}=K_{j}I_{j}+b_{j}+n_{j},$$

where j=1,2,3,4 is the number of pixels, $I_{j}$ denotes the intensity of the light passing through the pixelated polarizer filter, $K_{j}$ and $b_{j}$ are the scale factor and the bias, respectively, and $n_{j}$ is the measurement noise.

### 3.2. Installation Error of the Pixelated Polarizer Array

As shown in Figure 4, a polarization measuring unit is composed of a 2 × 2 array of CCD pixels. The pixelated polarizer is the same size as the CCD pixel. Ideally, during the process of integrating the pixelated polarizer array with the CCD camera, the polarizers will coincide well with the CCD pixels. However, there will be a certain installation error due to the limitation of alignment accuracy. For each CCD pixel, it will receive linearly polarized light passing through polarizers that have overlap with the CCD pixel, so Equation (1) to describe the response of the CCD pixels is not accurate. The installation errors mainly include angular misalignment and translation deviation, and the modeling process of the installation errors is described blow.

**Figure 5:**
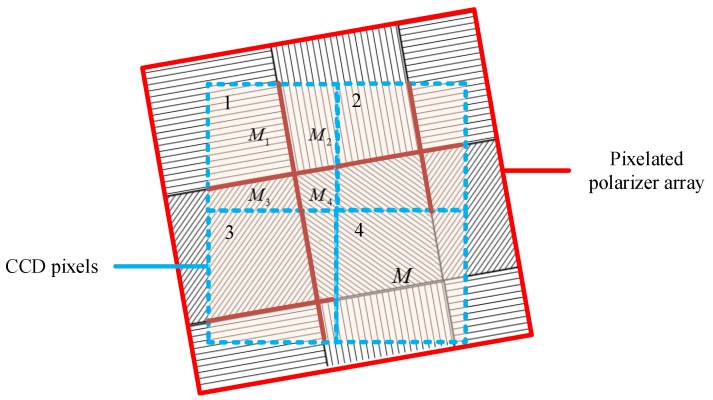
Schematic representation of the installation error of pixelated polarizer array.

Each CCD pixel receives linearly polarized light in four directions, and we calculate the response intensity of each CCD pixel by weighting its responses to linearly polarized light passing through four polarizers. The weight is determined by the area size of each polarizer overlapped with the CCD pixel. Suppose the area of each CCD pixel is *M*, and the area of CCD pixel overlapped with four polarizers is $M_{1}$, $M_{2}$, $M_{3}$ and $M_{4}$, respectively. Assuming the incident light is partially polarized light, with the total light intensity *I* and the degree of polarization *d*. For CCD pixel 1 in Figure 5, the intensity of light passing through the polarizers can be described by the following equation:

(16) $$\begin{array}{c} I_{1}=\frac{M_{1}I}{2M}\left[1+d\cos\left(2\phi -2\alpha_{1}\right)\right]+\frac{M_{2}I}{2M}\left[1+d\cos\left(2\phi -2\alpha_{2}\right)\right]\\ +\frac{M_{3}I}{2M}\left[1+d\cos\left(2\phi -2\alpha_{3}\right)\right]+\frac{M_{4}I}{2M}\left[1+d\cos\left(2\phi -2\alpha_{4}\right)\right]\\ =\frac{1}{2}I\left[1+k_{1}d\cos\left(2\phi -2\left(\alpha_{1}-\varepsilon_{1}\right)\right)\right]. \end{array}$$

The intensity of light passing through the polarizers for CCD pixels in one unit cell can be obtained:

(17) $$I_{j}=\frac{1}{2}I\left[1+k_{j}d\cos\left(2\phi -2\left(\alpha_{j}-\varepsilon_{j}\right)\right)\right],$$

where $\alpha_{1}$, $\alpha_{2}$, $\alpha_{3}$ and $\alpha_{4}$ are 0${}^{\circ }$, 90${}^{\circ }$, 45${}^{\circ }$ and 135${}^{\circ }$, respectively.

Substituting Equation (17) into Equation (15), the response function containing error parameters can be obtained:

(18) $$f_{j}=\frac{K_{j}}{2}I\left[1+k_{j}d\cos\left(2\phi -2\left(\alpha_{j}-\varepsilon_{j}\right)\right)\right]+b_{j}+n_{j}.$$

### 3.3. Calibration Method

Based on the analysis in Section 3.1 and Section 3.2, the calibration of our sensor is divided into two steps: the first step is to calibrate response error parameters, and the second step is the calibration of installation error of the pixelated polarizer array.

In order to estimate the linear error parameters $K_{j}$ and $b_{j}$ of the camera, we use a natural light source as input and record the response of the CCD camera at different light intensities. Then, the parameters can be determined by minimizing the following objective function:

(19) $$\min\sum_{i}{\left(K_{j}I_{j}^{i}+b_{j}-f_{j}^{i}\right)}^{2},$$

where *i* is the number of the measurement, and it should be greater than the number of parameters that need to be calibrated. $I_{j}^{i}$ and $f_{j}^{i}$ are the input light intensity and response of CCD pixel in the *i*th measurement, respectively.

After calibration of CCD response error parameters, we can estimate the intensity of light passing through the polarizers according to the response of the CCD camera. On the basis of the first step, the measurement equation including the installation errors can be expressed as:

(20) $$\tilde{A}\tilde{Q}=\tilde{F},$$

where

$$\tilde{A}=\left[\begin{array}{ccc} k_{1}\cos2\left(\alpha_{1}-\varepsilon_{1}\right) & k_{1}\sin2\left(\alpha_{1}-\varepsilon_{1}\right) & 1\\ k_{2}\cos2\left(\alpha_{2}-\varepsilon_{2}\right) & k_{2}\sin2\left(\alpha_{2}-\varepsilon_{2}\right) & 1\\ k_{3}\cos2\left(\alpha_{3}-\varepsilon_{3}\right) & k_{3}\sin2\left(\alpha_{3}-\varepsilon_{3}\right) & 1\\ k_{4}\cos2\left(\alpha_{4}-\varepsilon_{4}\right) & k_{4}\sin2\left(\alpha_{4}-\varepsilon_{4}\right) & 1 \end{array}\right],\tilde{Q}=\frac{1}{2}\left[\begin{array}{c} I\tilde{d}\cos2\tilde{\phi }\\ I\tilde{d}\sin2\tilde{\phi }\\ I \end{array}\right],\tilde{F}=\left[\begin{array}{c} \left(f_{1}-b_{1}\right)/K_{1}\\ \left(f_{2}-b_{2}\right)/K_{2}\\ \left(f_{3}-b_{3}\right)/K_{3}\\ \left(f_{4}-b_{4}\right)/K_{4} \end{array}\right].$$

The least square estimation of $\tilde{Q}$ is given by:

(21) $$\tilde{Q}={\left({\tilde{A}}^{T}\tilde{A}\right)}^{-1}{\tilde{A}}^{T}\tilde{F}.$$

Then,

(22) $$\tilde{\phi }=\frac{1}{2}\arctan\left(\frac{{\tilde{q}}_{2}}{{\tilde{q}}_{1}}\right),\;\tilde{d}=\frac{\sqrt{{\tilde{q}}_{1}^{2}+{\tilde{q}}_{2}^{2}}}{{\tilde{q}}_{3}},$$

where ${\tilde{q}}_{1}$, ${\tilde{q}}_{2}$ and ${\tilde{q}}_{3}$ are elements of $\tilde{Q}$.

In the calibration process, a high precision turntable is used to get the rotation angle of the adjacent two positions. The sensor is fixed on the precision turntable. Then, the precise rotation angle of the sensor can be achieved. Theoretically, the relative change of the polarization angle of incident light is equal to the rotation angle of the turntable. Relying on this precision turntable, an iterative least square method is employed to estimate parameters of installation error. Under a polarized light source, a total of N+1 measurements of polarization angle are obtained. This can be expressed as a function of the error parameters:

(23) $${\tilde{\phi }}_{m}=f\left(\varepsilon_{1},\varepsilon_{2},\varepsilon_{3},\varepsilon_{4},k_{1},k_{2},k_{3},k_{4}\right),m=0,1,2,\ldots ,N.$$

Define the following error equation:

(24) $$r_{m}={\tilde{\phi }}_{m}-{\tilde{\phi }}_{0}-\delta {\tilde{\phi }}_{0}-\Delta \phi_{m},$$

where *m* represents the *m*th measurement, ${\tilde{\phi }}_{m}$ is the *m*th measurement of the polarization angle, ${\tilde{\phi }}_{0}$ and $\delta {\tilde{\phi }}_{0}$ are the measurement of the polarization angle and its measurement error at the initial position, respectively. $\Delta \phi_{m}$ is the rotation angle of the turntable, namely, the benchmark of the relative change of the polarization angle. According to Equation (20), we can make:

(25) $$\begin{array}{c} k_{1}=1,\varepsilon_{1}=0. \end{array}$$

Based on the above analysis, seven independent variables have impacts on the result of $r_{k}$. Then, the vector of variables could be defined as follows:

(26) $$x={\left[\begin{array}{ccccccc} \varepsilon_{2} & \varepsilon_{3} & \varepsilon_{4} & k_{2} & k_{3} & k_{4} & \delta {\tilde{\phi }}_{0} \end{array}\right]}^{T}\;$$

Defining the following objective function, the optimal vector *x* can be obtained by minimizing the sum of error squares:

(27) $$\min f\left(x\right)=∥r(x)∥=\sum_{m=1}^{N}{r_{m}}^{2}\left(x\right).$$

As the objective function is a nonlinear function, the function r(x) is approximated by its linearization:

(28) $$r\left(x\right)\approx r\left(x_{n}\right)+J_{r}\left(x_{n}\right)\left(x-x_{n}\right),$$

where $J_{r}\left(x_{n}\right)$ is the Jacobian matrix, and its calculation method is detailed in the appendix. According to [36], the optimal solution could be computed as follows:

(29) $$x_{n+1}=x_{n}-{\left(J_{r}{\left(x_{n}\right)}^{T}J_{r}\left(x_{n}\right)\right)}^{-1}J_{r}{\left(x_{n}\right)}^{T}r\left(x_{n}\right),$$

where $x_{n+1}$ is the optimal solution after *n* iteration steps.

## 4. Results and Discussion

### 4.1. Calibration Results

The test system mainly consists of an integrating sphere uniform source (ISUS, FLD-03, Flight Technology), a linear polarizer and a high precision turntable. The integrating sphere uniform source is able to continuously provide uniform light of different intensity with a luminous uniformity of more than 98%. The natural light of the integrating sphere passes through the linear polarizer to obtain the standard linear polarized light. The PPL-compass is arranged on a high precision angle-dividing table, the repeating angular accuracy of which is better than 0.01${}^{\circ }$.

**Calibration of response error parameters:** In order to reduce the effect of external light source, the PPL-compass was placed in a sealed device. Uniform natural light was used as incident light. Then, the response error parameter of each pixel could be measured by changing the light intensity (from 0 to 1720 cd/m${}^{2}$, with intervals of about 60 cd/m${}^{2}$). At each light intensity step, five images were recorded by camera without any pixel overexposed during the increase of the intensity. Average of multiple images at each step were used to reduce the impact of measurement noise.

**Calibration of installation error parameters:** The PPL-compass was horizontally fixed on a precise angle dividing table, which has a total of 391 minimal rotating steps with each step representing 0.9207${}^{\circ }$. Then, the sensor was placed under polarized light source, and the rotation angle of the turntable is used as an external reference when calibrating installation error parameters. In the calibration process, the turntable started from 0${}^{\circ }$, and then steadily increased to 359.08${}^{\circ }$ at a step of 4.6036${}^{\circ }$ (5 minimal steps).

**Figure 6:**
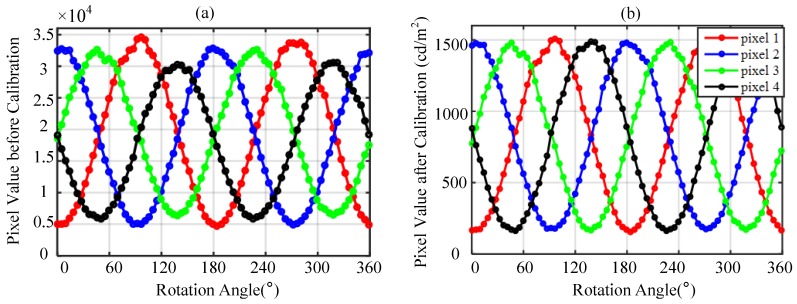
The values of four pixels in a polarization measurement unit fluctuate with the rotation of the turntable. (**a**) response curves before calibration; (**b**) response curves after calibration.

Figure 6a shows the response curves of four pixels in a polarization measurement unit before calibration with rotation of the turntable under a polarized light source. The response curves are approximately sinusoidal. Due to the response error and installation error, the amplitudes and offsets of four sine curves are not same, and the phase differences of sine signals are also not consistent with that of the polarity direction of polarizers. After calibration, the amplitudes and offsets of sine curve are basically the same, as shown in Figure 6b.

**Figure 7:**
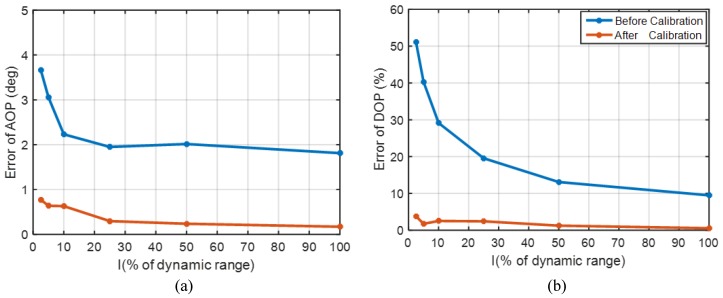
(**a**) the error of AOP as light intensity varies; (**b**) the error of DOP as light intensity varies.

Powell et al. [37] proposed another calibration formulation for pixelated polarized light sensor using the Muller matrix approach. This work evaluates a scalar and matrix calibration derived from a mathematical model of the polarimeter behavior. Our calibration method consists of two steps, and the first step is consistent with the calibration process of Powell based on the single pixel model. The primary difference lies in the different error model of the sensor. We assume that the polarizer does not exactly correspond to the CCD pixel and introduce the installation error parameters. As a consequence, each pixel gets contributions from neighboring pixels. In the second step of calibration, we estimate installation error parameters with the relative rotation angle of turntable under a polarized light source. Figure 7 shows improvements in the accuracy of and angle of polarization (AOP) and degree of polarization (DOP) for different intensities of light. The maximum intensity was set as high as possible without saturating any pixels at any angle of the polarizer. The remaining intensities were set at 50%, 25%, 10%, 5% and 2.5% of the maximum intensity. At each light intensity, the turntable started from 0${}^{\circ }$, and then steadily increased to 359.08${}^{\circ }$ at a step of 4.6036${}^{\circ }$ (five minimal steps). For a polarization measurement unit, the standard deviation of AOP error after calibration at maximum and minimum illumination is 0.17${}^{\circ }$ and 0.77${}^{\circ }$, respectively. The maximum RMS (Root Mean Square) error after calibration for DOP at maximum and minimum illumination is 0.5% and 3.8%, respectively.

**Figure 8:**
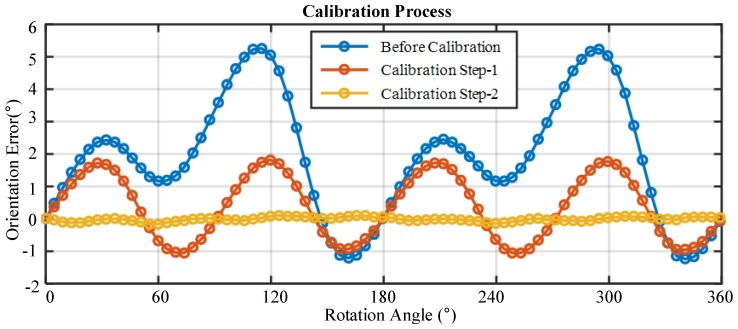
Orientation errors in the calibration process.

Taking the initial heading angle of the system as reference, the difference between the output of the PPL-compass and the rotation angle of the turntable is defined as the orientation error. Our orientation method make use of the AOP information of all the polarization measurement units. The orientation error in the calibration process is shown in Figure 8 and Table 1. The standard deviation of orientation error before calibration is 1.84${}^{\circ }$. After the calibration of response error parameters, the error curve is approximately sinusoidal, and the standard deviation of error is 0.99${}^{\circ }$. After the installation error parameters are calibrated, the orientation error improves greatly, and the standard deviation of the orientation error under standard polarized light reduces to 0.06${}^{\circ }$. Meanwhile, the maximum and average of orientation error also experience a great improvement, reducing to 0.15${}^{\circ }$ and −0.01${}^{\circ }$, respectively. The same experimental process was carried out three times, and similar results were obtained. These results show that our error model is accurate and the proposed calibration method is effective.

### 4.2. Outdoor Orientation Experiment

In order to verify the heading accuracy of PPL-compasses in outdoor environments, the orientation experiment was carried out on 28 May 2017 in Changsha. The angle-dividing table starts from 0${}^{\circ }$, and then steadily increased to 359.08${}^{\circ }$ at a step of 9.2072${}^{\circ }$ (10 minimal steps). At each position, one image was collected by a CCD camera, before the data acquisition PPL-compass had to be leveled by a leveling screw.

**Figure 9:**
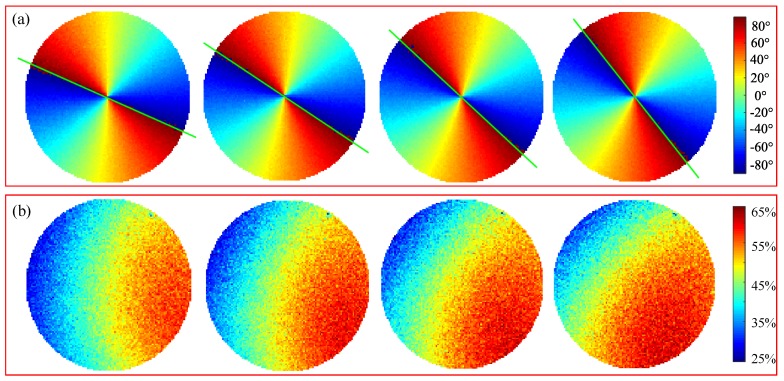
Skylight polarization patterns at four adjacent positions. (**a**) angle of polarization; (**b**) degree of polarization.

The polarization patterns of the sky were calculated by Equation (22). Figure 9 shows the skylight polarization pattern measured in the orientation experiment. Figure 9a shows the angle of polarization patterns of four adjacent positions, and Figure 9b is the corresponding degree of polarization patterns. Comparing the results of four frames, it is obvious that the polarization pattern rotates with the rotation of the turntable, and the polarization angle of pixels in a solar meridian line is a positive or negative 90${}^{\circ }$. The green line in the graph represents the projection of the solar meridian in the camera coordinate frame. The actual measurements of polarization angle patterns in a clear sky agree well with the single scattering Rayleigh model, that is, the E-vector of the scattered light is perpendicular to the scattering plane. Unlike the theoretical skylight polarization pattern, of which the maximum degree is 100%, the maximum degree of the polarization pattern in the actual orientation experiment is about 65%. Horvath et al. [38] showed that multiple scattering of light leads to depolarization and that the multiple scattering effects are greatly affected by atmospheric turbidity. Although the degree of polarization in actual measurement is lower than that of the single scattering Rayleigh model, our orientation method described in Section 2.3 mainly uses the AOP pattern to estimate the heading angle.

In outdoor tests, the calibration parameters obtained from indoor tests are used to calculate the output of PPL-compass. Then, the results are compared with the rotation angle of the turntable to evaluate heading accuracy of the sensor in outdoor environments. We compare the heading accuracy of our method detailed in this paper with the other two methods that are often used. Single point method [34] uses polarization information of incident light of only a specific direction, and we select pixels of images that point to the zenith direction to calculate the solar meridian. The line detection method [20] first extracts the pixels of which angle of polarization are close to negative and positive 90${}^{\circ }$, and then uses the Hough transform method to extract the solar meridian line.

**Figure 10:**
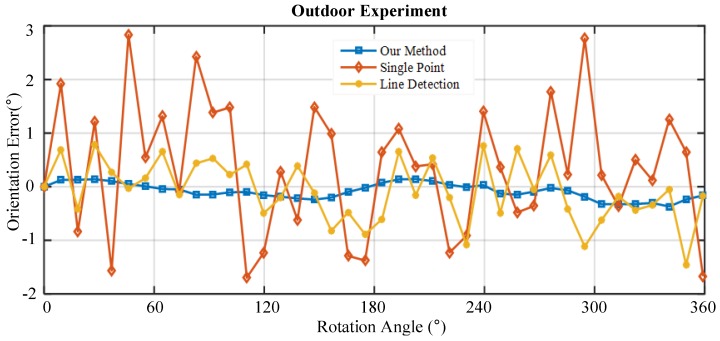
Comparison of three orientation methods.

The orientation error of three methods is shown in Figure 10 and Table 2. Our method achieves the best orientation results, the standard deviation of which is 0.15${}^{\circ }$. The maximum and the mean of heading error are 0.37${}^{\circ }$ and −0.08${}^{\circ }$, respectively. By contrast, the standard deviation of single point methods and line detection methods are 1.22${}^{\circ }$ and 0.57${}^{\circ }$, respectively, which are much greater than that of our method. Since the single point method only take into account the polarization information of incident light from one certain direction, it is most susceptible to noise interference. The line detection method overcomes the interference of the noise to a certain extent, but does not utilize the polarization information of all the pixels. Our method makes full use of polarization information of all the pixels, so it achieves the best heading accuracy.

Table 3 shows the experimental results of PPL-compasses at different solar elevation angles and different weather conditions. On a sunny day, the orientation accuracy of the sensor increases with the decrease of the solar elevation angle, and the standard deviation of orientation error is $0.15^{\circ }$ at sunset. On a cloudy day, the orientation accuracy of the sensor is worse than that of a clear day at the same solar elevation. Compared with the single-point based navigation sensor [39], our sensor achieves better orientation results due to the utilization of more abundant polarization information. It is recommended to use a polarized light compass for navigation in the morning and evening.

## 5. Conclusions

In this paper, a novel bio-inspired polarization sensor that mainly consists of a CCD camera, a pixelated polarizer array and a wide-angle lens is proposed. The measurement method of a skylight polarization pattern and the orientation method are presented. In the calibration process, we first establish its error model, including the response error of CCD pixels and the installation error of the pixelated polarizer array. Then, a calibration method based on iterative least squares estimation is put forward. After calibration, the maximum error of indoor orientation is 0.15${}^{\circ }$, and the standard deviation of the orientation error is about 0.06${}^{\circ }$, which proves the accuracy of the proposed error model and the validity of the calibration method. In outdoor experiments, the calibration parameters are used to calculate the output of PPL-compass, and three orientation methods are compared. The orientation accuracy of our method are better than those of the line detection method and the single point method because it takes full advantage of the information of all the pixels. The orientation accuracy of the sensor increases with the decrease of the solar elevation angle, and the standard deviation of orientation error is $0.15^{\circ }$ at sunset. The experimental results show that the proposed PPL-compass, which has the advantages of having a small size, being light weight and having a high sampling frequency, can be used in outdoor autonomous navigation.

## Figures and Tables

**Table 1:** Angle estimation errors of the indoor calibration process.

Calibration Process	Max Error (${}^{\circ }$)	Average Error (${}^{\circ }$)	Std Error (${}^{\circ }$)
Before Calibration	5.19	1.88	1.84
Calibration Step 1	1.82	0.36	0.99
Calibration Step 2	0.15	−0.01	0.06

**Table 2:** Angle estimation errors of three orientation methods.

Orientation Method	Max Error (${}^{\circ }$)	Average Error (${}^{\circ }$)	Std Error (${}^{\circ }$)
Single Point	2.84	0.35	1.22
Line Detection	1.46	−0.08	0.57
Our Method	0.37	−0.08	0.15

**Table 3:** Angle estimation errors at different solar elevation angles and different weather conditions.

Experiment Number	Weather	Solar Elevation Angle (${}^{\circ }$)	Std Error (${}^{\circ }$)
1	sunny	−0.5	0.15
2	sunny	5.5	0.32
3	sunny	9.1	0.43
4	sunny	13.5	0.61
5	sunny	18.2	0.87
6	cloudy	0.2	0.32

## Appendix A. Calculation of the Jacobian Matrix

The Jacobian matrix $J_{r}\left(x\right)$ can be expressed as follows:

(A1) $$J_{r}\left(x\right)={\left[\begin{array}{cccc} J_{r_{1}}{\left(x\right)}^{T} & J_{r_{2}}{\left(x\right)}^{T} & \cdots & J_{r_{N}}{\left(x\right)}^{T} \end{array}\right]}^{T}$$

According to Equation (24), we have:

(A2) $$J_{r_{m}}\left(x\right)=\left[\begin{array}{cc} J_{{\tilde{\phi }}_{m}}\left(x\left(1:6\right)\right) & -1 \end{array}\right]=\left[\begin{array}{cc} \frac{\partial {\tilde{\phi }}_{m}}{{\partial x(1:6)}^{T}} & -1 \end{array}\right].$$

For the calculation of $J_{{\tilde{\phi }}_{m}}\left(x\left(1:6\right)\right)$, the following intermediate variables are defined:

(A3) $${\tilde{A}}^{T}\tilde{A}=\left[\begin{array}{ccc} \sum_{j=1}^{4}a_{j1}^{2} & \sum_{j=1}^{4}a_{j1}a_{j2} & \sum_{j=1}^{4}a_{j1}\\ \sum_{j=1}^{4}a_{j1}a_{j2} & \sum_{j=1}^{4}a_{j2}^{2} & \sum_{j=1}^{4}a_{j2}\\ \sum_{j=1}^{4}a_{j1} & \sum_{j=1}^{4}a_{j2} & 4 \end{array}\right]=\left[\begin{array}{ccc} R_{1} & R_{2} & R_{3}\\ R_{2} & R_{4} & R_{5}\\ R_{3} & R_{5} & 4 \end{array}\right]=R,$$

(A4) $${\tilde{A}}^{T}\tilde{F}=\left[\begin{array}{c} \sum_{j=1}^{4}a_{j1}f_{j}\\ \sum_{j=1}^{4}a_{j2}f_{j}\\ \sum_{j=1}^{4}f_{j} \end{array}\right]=\left[\begin{array}{c} G_{1}\\ G_{2}\\ G_{3} \end{array}\right]=G.$$

The vector $\tilde{Q}$ can be expressed as follows:

(A5) $$\tilde{Q}=R^{-1}G=\frac{1}{T_{4}}{\left[\begin{array}{ccc} T_{1} & T_{2} & T_{3} \end{array}\right]}^{T},$$

where

$$\begin{array}{c} T_{1}=G_{1}\left(4R_{4}-R_{5}^{2}\right)+G_{2}\left(R_{3}R_{5}-4R_{2}\right)+G_{3}\left(R_{2}R_{5}-R_{3}R_{4}\right)\\ T_{2}=G_{1}\left(R_{3}R_{5}-4R_{2}\right)+G_{2}\left(4R_{1}-R_{3}^{2}\right)+G_{3}\left(R_{2}R_{3}-R_{1}R_{5}\right)\\ T_{3}=G_{1}\left(R_{2}R_{5}-R_{3}R_{4}\right)+G_{2}\left(R_{2}R_{3}-R_{1}R_{5}\right)+G_{3}\left(R_{1}R_{4}-R_{2}^{2}\right)\\ T_{4}=4R_{1}R_{4}+2R_{2}R_{3}R_{5}-R_{1}R_{5}^{2}-R_{3}^{2}R_{4}-4R_{2}^{2}. \end{array}$$

We calculate the derivatives of ${\tilde{\phi }}_{m}$ with respect to $\tilde{Q}$:

(A6) $$J_{{\tilde{\phi }}_{m}}\left(\tilde{Q}\right)=\frac{1}{2({{\tilde{q}}_{1}}^{2}+{{\tilde{q}}_{2}}^{2})}\left[\begin{array}{ccc} -{\tilde{q}}_{2} & {\tilde{q}}_{1} & 0 \end{array}\right].$$

The derivatives of $\tilde{Q}$ with respect to *T* are as follows:

(A7) $$J_{\tilde{Q}}\left(T\right)=\frac{1}{T_{4}^{2}}\left[\begin{array}{cccc} T_{4} & 0 & 0 & -T_{1}\\ 0 & T_{4} & 0 & -T_{2}\\ 0 & 0 & T_{4} & -T_{3} \end{array}\right].$$

*T* is a function of *R* and *G*, and define the following vector:

(A8) $$D={\left[\begin{array}{cccccccc} R_{1} & R_{2} & R_{3} & R_{4} & R_{5} & G_{1} & G_{2} & G_{3} \end{array}\right]}^{T}.$$

Then, $J_{T}\left(D\right)$ and $J_{D}\left(x(1:6)\right)$ are computed as follows:

(A9) $$\begin{array}{c} J_{T}\left(D\right)=\left[\begin{array}{cc} P_{1} & P_{2}\\ P_{3} & P_{4} \end{array}\right],\\ P_{1}=\left[\begin{array}{cccc} 0 & -4G_{2}+G_{3}R_{5} & G_{2}R_{5}-G_{3}R_{4} & 4G_{1}-G_{3}R_{3}\\ 4G_{2}-G_{3}R_{5} & -4G_{1}+G_{3}R_{3} & G_{1}R_{5}-2G_{2}R_{3}+G_{3}R_{2} & 0 \end{array}\right],\\ P_{2}=\left[\begin{array}{cccc} -2G_{1}R_{5}+G_{2}R_{3}+G_{3}R_{2} & 4R_{4}-R_{5}^{2} & R_{3}R_{5}-4R_{2} & R_{2}R_{5}-R_{3}R_{4}\\ G_{1}R_{3}-G_{3}R_{1} & R_{3}R_{5}-4R_{2} & 4R_{1}-R_{3}^{2} & R_{2}R_{3}-R_{1}R_{5} \end{array}\right],\\ P_{3}=\left[\begin{array}{cccc} -G_{2}R_{5}+G_{3}R_{4} & G_{1}R_{5}+G_{2}R_{3}-2G_{3}R_{2} & -G_{1}R_{4}+G_{2}R_{2} & -G_{1}R_{3}+G_{3}R_{1}\\ 4R_{4}-R_{5}^{2} & 2R_{3}R_{5}-8R_{2} & 2R_{2}R_{5}-2R_{3}R_{4} & 4R_{1}-R_{3}^{2} \end{array}\right],\\ P_{4}=\left[\begin{array}{cccc} G_{1}R_{2}-G_{2}R_{1} & R_{2}R_{5}-R_{3}R_{4} & R_{2}R_{3}-R_{1}R_{5} & R_{1}R_{4}-R_{2}^{2}\\ 2R_{2}R_{3}-2R_{1}R_{5} & 0 & 0 & 0 \end{array}\right], \end{array}$$

(A10) $$J_{D}\left(x(1:6)\right)=\left[\begin{array}{cccccc} 2k_{2}^{2}\sin4\theta_{2} & 2k_{3}^{2}\sin4\theta_{3} & 2k_{4}^{2}\sin4\theta_{4} & 2k_{2}\cos^{2}2\theta_{2} & 2k_{3}\cos^{2}2\theta_{3} & 2k_{4}\cos^{2}2\theta_{4}\\ -2k_{2}^{2}\cos4\theta_{2} & -2k_{3}^{2}\cos4\theta_{3} & -2k_{4}^{2}\cos4\theta_{4} & k_{2}\sin4\theta_{2} & k_{3}\sin4\theta_{3} & k_{4}\sin4\theta_{4}\\ 2k_{2}\sin2\theta_{2} & 2k_{3}\sin2\theta_{3} & 2k_{4}\sin2\theta_{4} & \cos2\theta_{2} & \cos2\theta_{3} & \cos2\theta_{4}\\ -2k_{2}^{2}\sin4\theta_{2} & -2k_{3}^{2}\sin4\theta_{3} & -2k_{4}^{2}\sin4\theta_{4} & 2k_{2}\sin^{2}2\theta_{2} & 2k_{3}\sin^{2}2\theta_{3} & 2k_{4}\sin^{2}2\theta_{4}\\ -2k_{2}\cos2\theta_{2} & -2k_{3}\cos2\theta_{3} & -2k_{4}\cos2\theta_{4} & \sin2\theta_{2} & \sin2\theta_{3} & \sin2\theta_{4}\\ 2I_{2}k_{2}\sin2\theta_{2} & 2I_{3}k_{3}\sin2\theta_{3} & 2I_{4}k_{4}\sin2\theta_{4} & I_{2}\cos2\theta_{2} & I_{3}\cos2\theta_{3} & I_{4}\cos2\theta_{4}\\ -2I_{2}k_{2}\cos2\theta_{2} & -2I_{3}k_{3}\cos2\theta_{3} & -2I_{4}k_{4}\cos2\theta_{4} & I_{2}\sin2\theta_{2} & I_{3}\sin2\theta_{3} & I_{4}\sin2\theta_{4}\\ 0 & 0 & 0 & 0 & 0 & 0 \end{array}\right],$$

where $\theta_{2}=\alpha_{2}-\varepsilon_{2},\;\theta_{3}=\alpha_{3}-\varepsilon_{3},\;\theta_{4}=\alpha_{4}-\varepsilon_{4}$.

The gradient (row-vector in this case) of ${\tilde{\phi }}_{m}$ with respect to x(1:6) is obtained:

(A11) $$J_{{\tilde{\phi }}_{m}}\left(x\left(1:6\right)\right)=J_{{\tilde{\phi }}_{m}}\left(\tilde{Q}\right)J_{\tilde{Q}}\left(T\right)J_{T}\left(D\right)J_{D}\left(x(1:6)\right).$$
